# On the effect of uncertainty on personal vaccination decisions

**DOI:** 10.1002/hec.4405

**Published:** 2021-08-03

**Authors:** Christophe Courbage, Richard Peter

**Affiliations:** ^1^ Geneva School of Business Administration University of Applied Sciences Western Switzerland (HES‐SO) Geneva Switzerland; ^2^ Department of Finance University of Iowa Iowa City Iowa USA

**Keywords:** ambiguity, primary prevention, uncertainty, vaccination

## Abstract

This study investigates the effect of ambiguity on personal vaccination decisions. We first characterize the vaccination decision in the absence of ambiguity. We then show that uncertainty about the probability of side effects and the efficacy of the vaccine always reduces take‐up under ambiguity aversion. However, uncertainty about the underlying disease, being the probability of sickness or the probability of a severe course of disease, may either encourage or discourage vaccination. Our results are relevant for policy because reducing uncertainty associated with the vaccine always has the desired effect whereas reducing uncertainty associated with the disease may have unintended consequences.

## 1 INTRODUCTION

Vaccination is considered as one of the main tools to eradicate pandemics and other infectious diseases (WHO, 2013), calling for a better understanding of individual vaccination decisions. While vaccines reduce the individual's probability of becoming sick, they may entail side effects such as headaches, fevers, muscle and joint aches or even death as illustrated by the Covid‐19 pandemic (Centers for Disease Control and Prevention, 2021). Therefore, at the individual level, the decision to vaccinate implies a trade‐off between its costs and benefits (Crainich et al., 2019).

In many circumstances, people take vaccination decisions under uncertainty. For instance, the Covid‐19 pandemic has shown that people face limited or contradictory sources of information regarding the disease (de la Oliva et al., 2021). They are uncertain about the chance of being infected, the efficacy of the vaccine, for example, due to the evolution of new strains, and its side effects. All these factors are likely to affect people's behavior (Han et al., 2018). Since Ellsberg's (1961) famous paradox, it is well known that individuals are often ambiguity‐averse, in particular in medical decision‐making (Attema et al., 2018; Portnoy et al., 2011). Against this background, we analyze how ambiguity affects individual vaccination decisions.

Our work connects to the literature on self‐protection (see Ehrlich & Becker, 1972), which has largely addressed financial risks (Dionne & Eeckhoudt, 1985; Eeckhoudt & Gollier, 2005; Peter, 2021b) and health risks (Courbage & Rey, 2006; Peter, 2021a). Yet, vaccination is a binary choice whereas the classical model of self‐protection considers the optimal level of effort, and side effects need to be incorporated. We also extend the decision‐threshold framework, introduced by Pauker and Kassirer (1975). Threshold analysis is well‐rooted in medical decision‐making (Felder & Mayrhofer, 2018), but has hardly addressed preventive decisions (Courbage & Rey, 2016). Finally, we extend Berger et al.'s (2013) study of curative care under ambiguity to preventive care.

We first provide a characterization of the vaccination decision in the absence of ambiguity. We then show that uncertainty about the probability of side effects and the efficacy of the vaccine always reduce take‐up of the vaccine under ambiguity aversion. However, uncertainty about the underlying disease itself, being the probability of sickness or the probability of a severe course of disease, may either encourage or discourage vaccination. This is relevant for policy because reducing uncertainty associated with the vaccine always has the desired effects whereas reducing uncertainty associated with the disease may have unintended consequences.

## 2 THE MODEL

We consider an individual with a two‐argument von Neumann‐Morgenstern utility function over consumption and health denoted by *u*(*C*, *H*). *C* represents consumption of an aggregate good and *H* measures health. We assume for simplicity that health can be measured with a single variable. Both consumption and health are valued, *u*_*C*_ > 0 and *u*_*H*_ > 0.

The individual faces a binary risk of sickness. She becomes sick with probability *p* ∈ (0, 1) and stays healthy with probability (1 − *p*). Sickness lowers health and is accompanied by (uninsured) *medical e*xpenditures or lost wages. *C*_*i*_ and *H*_*i*_ denote consumption and health in the healthy state (*i* = *h*) and the sick state (*i* = *s*), with *C*_*s*_ < *C*_*h*_ and *H*_*s*_ < *H*_*h*_. The individual's expected utility is given by

$$U^{n}=pu\left(C_{s},H_{s}\right)+\left(1-p\right)u\left(C_{h},H_{h}\right)\;,$$

where superscript *n* is shorthand for “not vaccinated.”

A vaccine is available, and we model its effects along the lines of Crainich et al. (2019). The vaccine reduces the probability of sickness by *e* ∈ (0, *p*). We assume that the monetary cost of the vaccine is zero either because it is covered by insurance or because it is negligibly small relative to consumption. Vaccination introduces the risk of side effects. There is a probability *q* ∈ (0, 1) for the individual to experience complications of *c* > 0. We assume the risk of sickness and the risk of side effects to be independent.^1^ Expected utility is then given by

(1) $$U^{v}=\left(p-e\right)\left(qu\left(C_{s},H_{s}-c\right)+\left(1-q\right)u\left(C_{s},H_{s}\right)\right)\;+\left(1-p+e\right)\left(qu\left(C_{h},H_{h}-c\right)+\left(1-q\right)u\left(C_{h},H_{h}\right)\right)\;,\;$$

where superscript *v* is shorthand for “vaccinated.” *U* ^*v*^ is increasing in the effectiveness of the vaccine *e*, and decreasing in the probability and severity of side effects *q* and *c*.

## 3 THE VACCINATION DECISION

Vaccination is valuable if and only if *U* ^*v*^ ≥ *U* ^*n*^, so that the vaccine raises expected utility.^2^ We will distinguish the individual's risk attitude over health, which can be averse, neutral or loving depending on the sign of *u*_*HH*_, and her attitude over correlation between consumption and health, which can also be averse, neutral or loving, depending on the sign of *u*_*CH*_. Recent empirical evidence on these preference traits is mixed (see, e.g., Attema et al., 2019). The following result characterizes the vaccination decision, see the Online Appendix A for a proof.

Proposition 1

(i) *Individuals with u*_*HH*_ ≤ 0 *and u*_*CH*_ ≤ 0 *vaccinate if and only if p* ≤ *p* ^*^ *. The threshold p* ^*^ *is decreasing in the severity of side effects with p* ^*^ = 1 *for* $c\leq \overset{ˇ}{c}$ (*always vaccinate) and p* ^*^ = *e for* $c\leq \overset{ˇ}{c}$(*never vaccinate). For* $c\in \left(\overset{ˇ}{c},\hat{c}\right)$ *, p* ^*^ *is between e and* 1.
(ii) *Individuals with u*_*HH*_ ≥ 0 *and u*_*CH*_ ≥ 0 *vaccinate if and only if p* ≥ *p* ^*^ *. The threshold p* ^*^ *is increasing in the severity of side effects with p* ^*^ = *e for* $c\leq \hat{c}$ *(always vaccinate) and p* ^*^ = 1 *for* $c\geq \hat{c}$ *(never vaccinate). For* $c\in \left(\hat{c},\overset{ˇ}{c}\right)$ *, p* ^*^ *is between e and* 1.
(iii) *For individuals with u*_*HH*_ < 0 *and u*_*CH*_ > 0 *or with u*_*HH*_ > 0 *and u*_*CH*_ < 0*, both cases can occur. The threshold p* ^*^ *is either decreasing or increasing in c*.

If potential side effects are mild, people will always favor vaccination, and if potential side effects are severe, people will always prefer to remain unvaccinated. In reality, we observe both types of decisions. This is the case for intermediate severity levels of side effects, that is, for $c\in \left(\overset{ˇ}{c},\hat{c}\right)$ in case (*i*) and for $c\in \left(\hat{c},\overset{ˇ}{c}\right)$ in case (*ii*). Then, a threshold on the sickness probability separates people in favor from people against vaccination. Notice that the decision rule is the same for anybody whose utility function has the specified derivatives whereas the exact magnitude of $\hat{c}$ and $\overset{ˇ}{c}$ depends on preferences and thus on the particular utility function.

**FIGURE 1:**
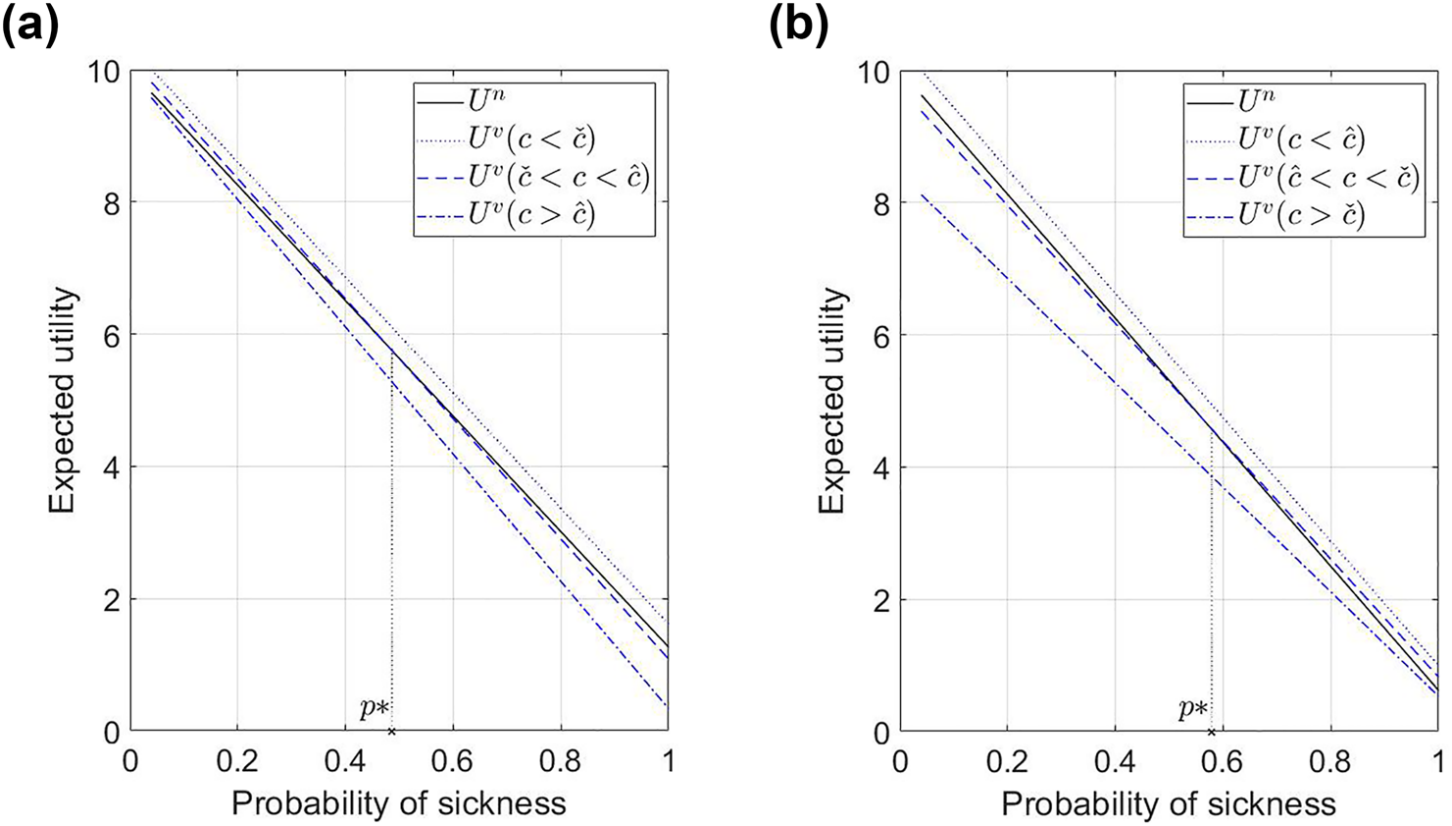
Illustration of Proposition 1. The blue lines represent expected utility at different levels of side effects. In case (*i*), for $c\in \left(\overset{ˇ}{c},\hat{c}\right)$, individuals vaccinate if *p* ≤ *p* ^*^; in case (*ii*), for $c\in \left(\hat{c},\overset{ˇ}{c}\right)$, individuals vaccinate if *p* ≥ *p* ^*^. (a) Case (*i*): *U* ^*v*^ intersects *U* ^*n*^ from above. (b) Case (*ii*): *U* ^*v*^ intersects *U* ^*n*^ from below

Figure 1 represents the two cases outlined in Proposition 1 graphically.^3^ In panel (a), an increase in the severity of side effects lowers *U* ^*v*^ but increases its slope so that it intersects *U* ^*n*^ from above. Therefore, the value of vaccination, defined by *V* = *U* ^*v*^ − *U* ^*n*^, is decreasing in *p* and people vaccinate if and only if *p* ≤ *p* ^*^. In panel (b), an increase in the severity of side effects lowers *U* ^*v*^ but now decreases its slope so that it intersects *U* ^*n*^ from below. The value of vaccination is now increasing in *p* and people vaccinate if and only if *p* ≥ *p* ^*^.

Intuition for this distinction can be derived in the spirit of harms disaggregation (see Eeckhoudt et al., 2007). If the individual is health risk‐ and correlation‐averse, she prefers to disaggregate harms and would rather allocate side effects to the healthy state than the sick state,

$$u\left(C_{h},H_{h}\right)+u\left(C_{s},H_{s}-c\right)\leq u\left(C_{h},H_{h}-c\right)+u\left(C_{s},H_{s}\right)\;.$$

Consequently, side effects increase the loss in utility from sickness. A higher probability of sickness therefore reduces expected utility by more for vaccinated than unvaccinated individuals, which explains the upper bar in Proposition 1(*i*) and why individuals favor vaccination for a low probability of sickness. If the individual is health risk‐ and correlation‐loving, she prefers to aggregate harms, matters are reversed and individuals favor vaccination for a high probability of sickness.

Proposition 1 can also provide some insights on positive externalities caused by vaccination. As more and more people are vaccinated, the probability of others getting sick is reduced. This will discourage some unvaccinated individuals from getting vaccinated in case (*ii*) but may surprisingly encourage other unvaccinated individuals to get vaccinated in case (*i*).

## 4 AMBIGUITY

We will now investigate how uncertainty affects the individual's vaccination decision. Some sources of uncertainty are associated with the vaccine while others are associated with the disease itself. In the first case, we will consider uncertainty over the probability of side effects and the efficacy of the vaccine. In the second case, we will consider uncertainty over the probability of sickness and the probability of the severity of sickness. This encompasses the main uncertainties currently experienced in the Covid‐19 crisis.

We focus on subjective beliefs because people may simply perceive uncertainty, especially when it comes to newly developed vaccines and new pandemics, for personal reasons including lack of information, limited trust in research, the government or the healthcare system, or due to a general sentiment of vaccine skepticism.^4^ However, our model also accommodates objective uncertainty such as scant scientific evidence. Uncertain probabilities are commonly referred to as ambiguity (see Ellsberg, 1961), and recent evidence suggests that people are more pessimistic in medical decisions under ambiguity than under risk, especially for health losses (see Attema et al., 2018). We use Ghirardato et al.'s (2004) *α*‐maxmin expected utility model to incorporate ambiguity aversion. In this model, the individual evaluates uncertain prospects via a weighted average over the worst case and the best case. It contains Gilboa and Schmeidler's (1989) famous maxmin model as a special case, which is still one of the most widely used models of decision‐making under ambiguity.

Consider first the probability of side effects, and let priors be built by *ε*‐contamination around *q* (see Epstein & Wang, 1994). The individual is uncertain about the likelihood of side effects and considers an entire range Q=[q(1−ε),ε+q(1−ε)]. People assign a confidence weight of (1 − *ε*) to *q*, and *ε* measures the size of the range. Parameter *ε* is commonly interpreted as a measure of perceived ambiguity. Let *α* ∈ [0, 1] denote the individual's ambiguity aversion. Her perceived welfare from vaccination is now given by

$$U_{a}^{v}=\alpha \min_{q^{\prime }\in Q}U^{v}+\left(1-\alpha \right)\max_{q^{\prime }\in Q}U^{v}\;.$$

In the case of uncertainty about the efficacy of the vaccine, the individual considers the range of efficacy values E=[e(1−ε),ε+e(1−ε)] with ε<(p−e)/(1−e) for positive probabilities. Her perceived welfare is derived analogously. The following proposition summarizes the effects of uncertainty associated with the vaccine, see the Online Appendix C for a proof.

Proposition 2 *Under ambiguity aversion, uncertainty about the probability of side effects or the efficacy of the vaccine lowers p* ^*^ *if u*_*HH*_ ≤ 0 *and u*_*CH*_ ≤ 0*, and raises p* ^*^ *if u*_*HH*_ ≥ 0 *and u*_*CH*_ ≥ 0*. For u*_*HH*_ < 0 *and u*_*CH*_ > 0 *or u*_*HH*_ > 0 *and u*_*CH*_ < 0*, both cases are possible*.

Uncertainty associated with the vaccine has the intuitive effect that it reduces the perceived value of the vaccine and fewer people vaccinate. What is interesting is that it can be people at high risk or at low risk of contracting the disease who forego the shot, depending on their risk attitude over health and correlation.

Let us now analyze uncertainties associated with the disease and consider the probability of sickness first. Let P=[p(1−ε),ε+p(1−ε)] denote the range of priors with ε<(p−e)/p to ensure positive probabilities. The following proposition holds, see the Online Appendix D.

Proposition 3 *Under ambiguity aversion, uncertainty about the probability of disease always lowers p* ^*^.

Contrary to Proposition 2, uncertainty about the probability of disease can either discourage or encourage vaccination. If *u*_*HH*_ ≤ 0 and *u*_*CH*_ ≤ 0, a lower *p* ^*^ means that fewer people vaccinate, while if *u*_*HH*_ ≥ 0 and *u*_*CH*_ ≥ 0, a lower *p* ^*^ means that more people vaccinate. If *u*_*HH*_ < 0 and *u*_*CH*_ > 0 or *u*_*HH*_ > 0 and *u*_*CH*_ < 0, both effects are possible.

Finally, we extend the baseline model by allowing for different severity levels of the disease. Let *C*_ss_ < *C*_*s*_ and *H*_ss_ < *H*_*s*_ denote consumption and health if the individual is severely sick as abbreviated by subscript ss, and let *π* denote the probability of experiencing a severe course of disease. Let П = [*π*(1 − *ε*), *ε* + *π*(1 − *ε*)] denote the range of priors with *ε* < (*π* − *e*)/*π*. The following proposition holds, see the Online Appendix E.

Proposition 4 *Under ambiguity aversion, uncertainty about the probability of a severe course of the disease lowers p* ^*^ *if u*_*HH*_ ≥ 0 *and u*_*CH*_ ≥ 0*; if u*_*HH*_ ≤ 0 *and u*_*CH*_ ≤ 0*, it raises p* ^*^ *if q is below an endogenous threshold* $\tilde{q}$ *and lowers it otherwise. For u*_*HH*_ < 0 *and u*_*CH*_ > 0 *or u*_*HH*_ > 0 *and u*_*CH*_ < 0*, both cases are possible*.

The prevailing case is that uncertainty about the probability of experiencing a severe course of disease encourages vaccination, especially when the probability of side effects is small, but as in the case of uncertainty about the probability of disease, the contrary may occur.

## 5 CONCLUSION

In the context of the ongoing Covid‐19 pandemic, people across the world have made or must still make a decision to be vaccinated or not in the presence of significant uncertainty. This study has investigated the effects of ambiguity on individual decisions to vaccinate. It reveals that such effects depend on the source of ambiguity, which has important policy perspectives. Indeed, our results show that reducing uncertainty associated with the vaccine will always encourage vaccination whereas reducing uncertainty associated with the disease may have unintended “side effects” and discourage vaccination for some individuals, depending on their risk preferences over health and correlation.

Various avenues for future research can be considered. First, our model relies on expected utility which has limited descriptive validity, including in the health domain (Bleichrodt et al., 2007). A natural extension would be to consider the rank‐dependent utility model (Quiggin, 1982) or reference‐dependent models (Kahneman & Tversky, 1979), which have better descriptive validity. Second, the theoretical results of our study lend themselves to empirical investigations, whether in the form of experimental work or quantitative analysis to investigate the decision to vaccinate under uncertainty. There is no doubt that the current Covid‐19 crisis should offer ample opportunities for such follow‐up studies.

## CONFLICT OF INTEREST

The authors have no conflicts of interests to disclose.

## Supporting information

Supporting Information S1

## ENDNOTES

^1^
Independence implies that individuals who experience side effects are no more or less likely to contract the disease later than those individuals without side effects.

^2^
We assume that individuals decide in favor of vaccination when indifferent.

^3^
The Online Appendix B provides the underlying parameter choices and utility functions.

^4^
These examples not only lead to increased uncertainty but could also reduce people's subjective belief about the efficacy of vaccination. In this case, our model predicts a lower take‐up of the vaccine.
