# Supplementary material for: On the effect of uncertainty on personal vaccination decisions
Source: Health Econ. 2021 Aug 3;30(11):2937–42. doi: 10.1002/hec.4405 (PMC9290645; doi:10.1002/hec.4405)
Supplement: Supplementary file 1 — Supporting Information S1 [file HEC-30-2937-s001.docx]

**Appendix A – Proof of Proposition 1**

Both $U^{v}$ and $U^{n}$ are linearly decreasing in $p$. Define the following auxiliary function:

$$f\left( c \right)=q\left[ u\left( C_{h},H_{h}-c \right)-u\left( C_{h},H_{h} \right) \right]+e\left[ u\left( C_{h},H_{h} \right)-u\left( C_{s},H_{s} \right) \right].$$

$f$ measures the difference between $U^{v}$ and $U^{n}$ when $p\to e$. It is strictly decreasing in $c$ with $f\left( 0 \right)=e\left[ u\left( C_{h},H_{h} \right)-u\left( C_{s},H_{s} \right) \right]>0$. Let $\hat{c}$ denote the unique zero of $f$ where it switches from positive to negative. Now define another auxiliary function:

$$g\left( c \right)=e\left[ qu\left( C_{h},H_{h}-c \right)+\left( 1-q \right)u\left( C_{h},H_{h} \right)-u\left( C_{s},H_{s} \right) \right]-\left( 1-e \right)q\left[ u\left( C_{s},H_{s} \right)-u\left( C_{s},H_{s}-c \right) \right].$$

$g$ measures the difference between $U^{v}$ and $U^{n}$ when $p\to1$. It is strictly decreasing in $c$ with $g\left( 0 \right)=e\left[ u\left( C_{h},H_{h} \right)-u\left( C_{s},H_{s} \right) \right]>0$. Let $\check{c}$ denote the unique zero of $g$ where it switches from positive to negative.

We distinguish between two cases. If $\check{c}<\hat{c}$, then $U^{v}>U^{n}$ for $c\leq\check{c}$ so that vaccination is preferred and $U^{v}<U^{n}$ for $c\geq\hat{c}$ so that remaining unvaccinated is preferred. For $c\in\left( \check{c},\hat{c} \right)$, $U^{v}$ is steeper in $p$ than $U^{n}$ because $U^{v}>U^{n}$ for $p\to e$ and $U^{v}<U^{n}$ for $p\to1$. We can then find a unique $p^{*}\in\left( e,1 \right)$ such that $U^{v}\geq U^{n}$ for $p\leq p^{*}$. The threshold $p^{*}$ is decreasing in $c$ because $U^{v}$ is lower the higher $c$ is and $U^{v}$ crosses $U^{n}$ from above. If $\hat{c}<\check{c}$ instead, then $U^{v}>U^{n}$ for $c\leq\hat{c}$ so that vaccination is preferred and $U^{v}<U^{n}$ for $c\geq\check{c}$ so that remaining unvaccinated is preferred. For $c\in\left( \hat{c},\check{c} \right)$, $U^{v}$ is shallower in $p$ than $U^{n}$ because $U^{v}<U^{n}$ for $p\to e$ and $U^{v}>U^{n}$ for $p\to1$. We can then find a unique $p^{*}\in\left( e,1 \right)$ such that $U^{v}\geq U^{n}$ for $p\geq p^{*}$. The threshold $p^{*}$ is now increasing in $c$ because $U^{v}$ is lower the higher $c$ is and $U^{v}$ crosses $U^{n}$ from below.^[[1]](#footnote-1)^

To determine whether $\hat{c}$ or $\check{c}$ is larger, we insert $\hat{c}$ into $g$ and determine the sign. We utilize $f\left( \hat{c} \right)=0$ and obtain the following:

$$g\left( \hat{c} \right)=\left( 1-e \right)q\left[ u\left( C_{h},H_{h} \right)-u\left( C_{h},H_{h}-\hat{c} \right)-u\left( C_{s},H_{s} \right)+u\left( C_{s},H_{s}-\hat{c} \right) \right].$$

We expand the square bracket to

$$\left[ u\left( C_{h},H_{h} \right)-u\left( C_{h},H_{h}-\hat{c} \right)-u\left( C_{s},H_{h} \right)+u\left( C_{s},H_{h}-\hat{c} \right) \right]$$

$$+\left[ u\left( C_{s},H_{h} \right)-u\left( C_{s},H_{h}-\hat{c} \right)-u\left( C_{s},H_{s} \right)+u\left( C_{s},H_{s}-\hat{c} \right) \right].$$

$u_{HH}\leq0$ and $u_{CH}\leq0$ ensure that both terms are non-positive so that $\check{c}\leq\hat{c}$, with a strict inequality if either $u_{HH}\leq0$ or $u_{CH}\leq0$ is strict. This shows result (*i*). $u_{HH}\geq0$ and $u_{CH}\geq0$ ensure that both terms are non-negative so that $\check{c}\geq\hat{c}$, with a strict inequality if either $u_{HH}\geq0$ or $u_{CH}\geq0$ is strict. This shows result (*ii*). If $u_{HH}<0$ and $u_{CH}>0$ or if $u_{HH}>0$ and $u_{CH}<0$, we cannot sign $g\left( \hat{c} \right)$ and both cases are possible, $\check{c}<\hat{c}$ with a threshold $p^{*}$ that is decreasing in $c$, or $\hat{c}<\check{c}$ with a threshold $p^{*}$ that is increasing in $c$. This shows result (*iii*).

**Appendix B – Parameters underlying Figure 1**

We set $C_{h}=100$, $C_{s}=50$, $H_{h}=1$, and $H_{s}=0.65$ for consumption and health in the two states of the world. For the vaccine, we assume $e=0.04$ and $q=0.25$ for the efficacy and probability of side effects. The utility function for panel (a) is $u\left( C,H \right)=-C^{-0.5}\cdot H^{-0.5}$, which satisfies $u_{C}>0$, $u_{H}>0,$ $u_{CC}<0$, $u_{HH}<0$ and $u_{CH}<0$ so that Proposition 1(*i*) applies. We choose three levels for the severity of side effects, $c=0, 0.12$ and $0.24$, and normalize the utility function so that $U^{n}$ and $U^{v}$ are between $0$ and $10$. For $c=0$, we obtain $U^{v}>U^{n}$, and for $c=0.24$, we obtain $U^{v}<U^{n}$ for all probabilities $p\in\left( e,1 \right)$. For $c=0.12$, the vaccination threshold is $p^{*}=0.48$, and people vaccinate if and only if $p\leq p^{*}$.

For panel (b), the utility function is $u\left( C,H \right)=\left( -0.5\cdot C^{-0.5}+0.5 \right)\cdot H^{4}$, which satisfies $u_{C}>0$, $u_{H}>0,$ $u_{CC}<0$, $u_{HH}>0$ and $u_{CH}>0$ so that Proposition 1(*ii*) applies. Notice that $u_{H}>0$ and $u_{HH}>0$ require $C\geq1$, which is given with our choice of parameters. We choose three levels for the severity of side effects, $c=0, 0.06$ and $0.24$, and normalize the utility function so that $U^{n}$ and $U^{v}$ are between $0$ and $10$. For $c=0$, we obtain $U^{v}>U^{n}$, and for $c=0.24$, we obtain $U^{v}<U^{n}$ for all probabilities $p\in\left( e,1 \right)$. For $c=0.06$, the vaccination threshold is $p^{*}=0.58$, and people vaccinate if and only if $p\geq p^{*}$.

**Appendix C – Proof of Proposition 2**

If $u_{HH}\leq0$ and $u_{CH}\leq0$, we know from Proposition 1 that the value of vaccination in the absence of ambiguity, $V=U^{v}-U^{n}$, is decreasing in $p$ with $V\left( p^{*} \right)=0$. If the probability of side effects is uncertain, we obtain

$$\min_{q^{'}\in Q} U^{v}=\left( p-e \right)\left( \left( \varepsilon+q\left( 1-\varepsilon\right) \right)u\left( C_{s},H_{s}-c \right)+\left( 1-q \right)\left( 1-\varepsilon\right)u\left( C_{s},H_{s} \right) \right)+\left( 1-p+e \right)\left( \left( \varepsilon+q\left( 1-\varepsilon\right) \right)u\left( C_{h},H_{h}-c \right)+\left( 1-q \right)\left( 1-\varepsilon\right)u\left( C_{h},H_{h} \right) \right)$$

and

$$\max_{q^{'}\in Q} U^{v}=\left( p-e \right)\left( q\left( 1-\varepsilon\right)u\left( C_{s},H_{s}-c \right)+\left( 1-q\left( 1-\varepsilon\right) \right)u\left( C_{s},H_{s} \right) \right)+\left( 1-p+e \right)\left( q\left( 1-\varepsilon\right)u\left( C_{h},H_{h}-c \right)+\left( 1-q\left( 1-\varepsilon\right) \right)u\left( C_{h},H_{h} \right) \right),$$

and therefore

$$U_{a}^{v}=\left( p-e \right)\left( \left( q+\varepsilon\left( \alpha-q \right) \right)u\left( C_{s},H_{s}-c \right)+\left( 1-q-\varepsilon\left( \alpha-q \right) \right)u\left( C_{s},H_{s} \right) \right)+\left( 1-p+e \right)\left( \left( q+\varepsilon\left( \alpha-q \right) \right)u\left( C_{h},H_{h}-c \right)+\left( 1-q-\varepsilon\left( \alpha-q \right) \right)u\left( C_{h},H_{h} \right) \right).$$

Ambiguity aversion prevails if $\alpha>q$ because then $U_{a}^{v}<U^{v}$ so that ambiguity makes the individual worse off. The value of vaccination under ambiguity is given by $V_{a}=U_{a}^{v}-U^{n}$ because expected utility when deciding to remain unvaccinated is unaffected by uncertainty over the probability of side effects. Under our assumptions on preferences, $V_{a}$ is also decreasing in $p$, which follows along the lines of the proof of Proposition 1. We then obtain

$$V_{a}\left( p^{*} \right)=U_{a}^{v}\left( p^{*} \right)-U^{n}\left( p^{*} \right)<U^{v}\left( p^{*} \right)-U^{n}\left( p^{*} \right)=V\left( p^{*} \right)=0,$$

so that ambiguity reduces $p^{*}$ and fewer people vaccinate. If $u_{HH}\geq0$ and $u_{CH}\geq0$, then $V_{a}$ is increasing in $p$. We obtain $V_{a}\left( p^{*} \right)<0$, which now implies that ambiguity raises $p^{*}$ but again fewer people vaccinate. If $u_{HH}<0$ and $u_{CH}>0$ or if $u_{HH}>0$ and $u_{CH}<0$, then $V_{a}$ can be decreasing or increasing in $p$ and both scenarios are possible. This shows Proposition 2. We omit the proof for uncertainty over the efficacy of the vaccine because it is very similar.

**Appendix D – Proof of Proposition 3**

If the probability of disease is uncertain, the individual’s expected utility with and without the vaccine are both affected. We obtain

$$U_{a}^{n}=\left( p+\varepsilon\left( \alpha-p \right) \right)u\left( C_{s},H_{s} \right)+\left( 1-p-\varepsilon\left( \alpha-p \right) \right)u\left( C_{h},H_{h} \right)$$

for expected utility under ambiguity without the vaccine and

$$U_{a}^{v}=\left( p+\varepsilon\left( \alpha-p \right)-e \right)\left( qu\left( C_{s},H_{s}-c \right)+\left( 1-q \right)u\left( C_{s},H_{s} \right) \right)+\left( 1-p-\varepsilon\left( \alpha-p \right)+e \right)\left( qu\left( C_{h},H_{h}-c \right)+\left( 1-q \right)u\left( C_{h},H_{h} \right) \right)$$

for expected utility under ambiguity with the vaccine. The value of vaccination under ambiguity, defined as $V_{a}=U_{a}^{v}-U_{a}^{n}$, can be rewritten as

$$V_{a}=V+\varepsilon\left( \alpha-p \right)q\cdot\left[ u\left( C_{h},H_{h} \right)-u\left( C_{h},H_{h}-c \right)-u\left( C_{s},H_{s} \right)+u\left( C_{s},H_{s}-c \right) \right]$$

where $V=U^{v}-U^{n}$ denotes the value of vaccination without ambiguity.

Then

$$\frac{\partial V_{a}}{\partial p}=\left( 1-\varepsilon\right)q\cdot\left[ u\left( C_{h},H_{h} \right)-u\left( C_{h},H_{h}-c \right)-u\left( C_{s},H_{s} \right)+u\left( C_{s},H_{s}-c \right) \right].$$

If the square bracket is negative, both $V$ and $V_{a}$ are decreasing in $p$, intersect at $p=\alpha$, and $V$ is steeper than $V_{a}$. So if $V$ is uniformly nonnegative, $V_{a}$ is as well and if $V$ is uniformly negative, so is $V_{a}$. The only interesting case is if $V$ changes sign at $p^{*}$. If this occurs in the region where ambiguity aversion prevails (i.e., $\alpha>p^{*}$), then $V_{a}\left( p^{*} \right)<0$ and ambiguity lowers $p^{*}$. If the square bracket is positive, both $V$ and $V_{a}$ are increasing in $p$, intersect at $p=\alpha$, and $V$ is steeper than $V_{a}$. Again, the only interesting case is if $V$ changes sign at $p^{*}$. If this occurs in the region where ambiguity aversion prevails (i.e., $\alpha>p^{*}$), then $V_{a}\left( p^{*} \right)>0$ and ambiguity lowers $p^{*}$.

**Appendix E – Proof of Proposition 4**

In the extended model, we have

$$U^{n}=p\left[ \pi u\left( C_{ss},H_{ss} \right)+\left( 1-\pi\right)u\left( C_{s},H_{s} \right) \right]+\left( 1-p \right)u\left( C_{h},H_{h} \right)$$

for the individual’s expected utility without vaccination and

$$U^{v}=\left( p-e \right)\pi\left( qu\left( C_{ss},H_{ss}-c \right)+\left( 1-q \right)u\left( C_{ss},H_{ss} \right) \right)+\left( p-e \right)\left( 1-\pi\right)\left( qu\left( C_{s},H_{s}-c \right)+\left( 1-q \right)u\left( C_{s},H_{s} \right) \right)+\left( 1-p+e \right)\left( qu\left( C_{h},H_{h}-c \right)+\left( 1-q \right)u\left( C_{h},H_{h} \right) \right)$$

for the individual’s expected utility with the vaccine. $U^{v}$ and $U^{n}$ are linearly decreasing in $p$, and one can use the same approach as in Appendix A to show that Proposition 1 still holds. Uncertainty over the probability of experiencing a severe course of disease results in expected utilities of $U_{a}^{n}$ and $U_{a}^{v}$ with $\pi$ replaced by $\pi+\varepsilon\left( \alpha-\pi\right)$ in $U^{v}$ and $U^{n}$. Ambiguity aversion requires $\alpha>\pi$ so that $U_{a}^{n}<U^{n}$ and $U_{a}^{v}<U^{v}$.

Direct computation shows that the value of vaccination under ambiguity is given by

$$V_{a}=V-\varepsilon\left( \alpha-\pi\right)\cdot\left[ \left( p-e \right)q\left( u\left( C_{s},H_{s}-c \right)-u\left( C_{s},H_{s} \right)-u\left( C_{ss},H_{ss}-c \right)+u\left( C_{ss},H_{ss} \right) \right)-e\left( u\left( C_{s},H_{s} \right)-u\left( C_{ss},H_{ss} \right) \right) \right],$$

where $V=U^{v}-U^{n}$ denotes the value of vaccination without ambiguity. The sign of the square bracket determines whether ambiguity increases or reduces the value of vaccination. We always have $u\left( C_{s},H_{s} \right)>u\left( C_{ss},H_{ss} \right)$; furthermore, if $u_{HH}\geq0$ and $u_{CH}\geq0$, then

$$u\left( C_{s},H_{s}-c \right)+u\left( C_{ss},H_{ss} \right)\leq u\left( C_{s},H_{s} \right)+u\left( C_{ss},H_{ss}-c \right),$$

and the square bracket is negative. As a result, $V_{a}\left( p^{*} \right)>0$, and ambiguity lowers $p^{*}$ because $u_{HH}\geq0$ and $u_{CH}\geq0$ ensure that $V_{a}$ is increasing in $p$. If $u_{HH}\leq0$ and $u_{CH}\leq0$, the square bracket can be positive or negative. Define

$$\tilde{q}=\text{min}\left\{ \frac{e}{p^{*}-e}\cdot\frac{u\left( C_{s},H_{s} \right)-u\left( C_{ss},H_{ss} \right)}{u\left( C_{s},H_{s}-c \right)-u\left( C_{s},H_{s} \right)-u\left( C_{ss},H_{ss}-c \right)+u\left( C_{ss},H_{ss} \right)},1 \right\};$$

then $V_{a}\left( p^{*} \right)>0$ for $q<\tilde{q}$, and ambiguity raises $p^{*}$ because $u_{HH}\leq0$ and $u_{CH}\leq0$ ensure that $V_{a}$ is decreasing in $p$. For $q>\tilde{q}$, we obtain $V_{a}\left( p^{*} \right)<0$ and ambiguity lowers $p^{*}$.

1. The knife-edge case $\check{c}=\hat{c}$ is trivial because either everybody vaccinates for $c\leq\hat{c}$ or nobody vaccinates for $c>\hat{c}$. There are no intermediate scenarios where some individuals vaccinate and others do not. [↑](#footnote-ref-1)
